# The Development of Urease Inhibitors: What Opportunities Exist for Better Treatment of *Helicobacter pylori* Infection in Children?

**DOI:** 10.3390/children4010002

**Published:** 2017-01-04

**Authors:** Sherif T. S. Hassan, Miroslava Šudomová

**Affiliations:** 1 Department of Natural Drugs, Faculty of Pharmacy, University of Veterinary and Pharmaceutical Sciences Brno, Palackého tř. 1946/1, 61242 Brno, Czech Republic; 2 Department of Applied Ecology, Faculty of Environmental Sciences, Czech University of Life Sciences Prague, Kamýcká 129, 165 21 Praha 6—Suchdol, Czech Republic; 3 Museum of the Brno Region, Museum of Literature in Moravia, Porta Coeli 1001, 66602 Předklášteří, Czech Republic; sudomova@post.cz

**Keywords:** urease inhibitors, *Helicobacter pylori* (*H. pylori*) infection, children, drug development, pharmacokinetics

## Abstract

Stomach infection with *Helicobacter pylori* (*H. pylori)* causes severe gastroduodenal diseases in a large number of patients worldwide. The *H. pylori* infection breaks up in early childhood, persists lifelong if not treated, and is associated with chronic gastritis and an increased risk of peptic ulcers and gastric cancer. In recent years, the problem of drug-resistant strains has become a global concern that makes the treatment more complicated and the infection persistent at higher levels when the antibiotic treatment is stopped. Such problems have led to the development of new strategies to eradicate an *H. pylori* infection. Currently, one of the most important strategies for the treatment of *H. pylori* infection is the use of urease inhibitors. Despite the fact that large numbers of molecules have been shown to exert potent inhibitory activity against *H. pylori* urease, most of them were prevented from being used in vivo and in clinical trials due to their hydrolytic instability, toxicity, and appearance of undesirable side effects. Therefore, it is crucial to focus attention on the available opportunities for the development of urease inhibitors with suitable pharmacokinetics, high hydrolytic stability, and free toxicological profiles. In this commentary, we aim to afford an outline on the current status of the use of urease inhibitors in the treatment of an *H. pylori* infection, and to discuss the possibility of their development as effective drugs in clinical trials.

## 1. Introduction

*Helicobacter pylori* (*H. pylori*) is a ubiquitous Gram-negative bacterium, which survives in the mucus layer overlying the gastric mucosa, and infects about 30% of children worldwide [1]. *H. pylori* colonizes the stomach and can induce diseases such as peptic ulcers, gastritis, and gastric cancer [2,3]. The pathogen persists in the stomach for decades and most infected children may never observe clinical symptoms, despite having chronic gastritis and 20–30% of those colonized by *H. pylori* may ultimately develop peptic ulcers [4,5]. Several treatment regimens, including triple therapy which consists of two antibiotics and a proton pump inhibitor (or ranitidine bismuth) administered over 7 days, have been shown to eradicate *H. pylori* effectively [6,7,8]. The most commonly used antibiotics are tetracycline, amoxicillin, imidazole (metronidazole or tinidazol), and macrolids (clarithromycin or azithromycin) [9,10,11]. Despite the efficacy of these regimens, several limitations exist, such as the lack of therapeutic compliance due to the incidence of adverse effects and the discomfort of multiple doses, and thus has the potential to lead to the development of drug-resistant strains [12,13,14]. Additionally, antibiotics such as amoxicillin and clarithromycin are known to be degraded by gastric acid. Therefore, it becomes necessary to use higher doses, which often results in an increase of gastrointestinal (GI) side effects, such as diarrhea, nausea, vomiting, bloating, and abdominal pain [15,16,17].

For these reasons, it has become necessary to search for alternative strategies to overcome such problems. In recent years, new treatment strategies have been developed to promote treatment efficacy and overcome the complications of drug-resistant strains and undesirable side effects, through the use of urease inhibitors.

## 2. The Role of Urease in *Helicobacter pylori* (*H. pylori*)

Urease (EC 3.5.1.5; urea amidohydrolase) is a nickel-containing enzyme that catalyzes the hydrolysis of urea to ammonia and carbamate; the latter decomposes spontaneously to produce another molecule of ammonia and carbon dioxide [18,19]. This enzyme is widely found in nature such as in plants, bacteria, fungi, and algae [20,21]. Urease plays an important role in the infection capabilities of *H. pylori*. It allows this pathogen to survive, grow, and multiply at the low pH of the stomach, spreading infection to the inner layers of gastroduodenal mucosa, resulting in gastritis and peptic ulceration, which in some cases leads to gastric cancer [22,23]. Urease constitutes 10–15% (*w*/*w*) of the total proteins produced by *H. pylori*, and presents in both the cytoplasmic and surface-associated forms [24]. Additionally, intracellular urease is responsible for cytoplasmic pH homeostasis, due to its enhanced release of ammonia into the periplasmic space [25]. Another report has described the presence of external urease as a result of “altruistic autolysis.” This is done by affecting a part of the bacterial population in response to the low pH environment with consecutive re-adsorption of the released enzyme on the outer cell membrane of intact bacteria, as an additional protective urease coat [26].

*H. pylori* urease has a specific macromolecular structure that differs from other bacterial ureases. For instance, *H. pylori* urease has been reported to consist of two monomers, namely the 26.5 kDa α-subunit and the 61.7 kDa β-subunit, that form 12 catalytic α–β heterodimers with a unique dodecameric ((α–β)3)4 architecture [27]. Moreover, the β-subunit was found to play a vital role in assembling the urease molecule, where its N- and C-terminal domains are bound tightly with neighboring α-subunits, producing trimers with threefold symmetry, while the extended C-terminal domain forms an off-surface α-helix and a terminal loop that links the adjacent β-subunits in a spherical supramolecular tetramer, in a head-to-tail manner [28,29]. Detailed crystallographic reports have revealed that the complex structure of *H. pylori* urease may give self-supporting protection from the inactivation of neighboring catalytic units [30,31]. It has been reported that the structure of the *H. pylori* urease active site is also different from other bacterial ureases, due to its characteristic flap motion and flexibility, which contributes to an unusually high enzyme affinity to the substrate [32].

Additionally, two reports have described that the UreI protein (responsible for the maturation of the enzyme) is an important component of the cytoplasmic membrane, which acts as a selective pH-gated urea channel, thus allowing the bacterium to quickly acclimate to an acidic pH due to the enhanced intracellular urease response [33,34].

## 3. Current Development of Urease Inhibitors

Over the past two decades, extensive studies have been conducted on natural products and synthetic or semisynthetic drugs, in order to evaluate their potential inhibitory effect against *H. pylori* urease. Interestingly, a large number of these molecules were found to possess potent in vitro inhibitory properties against urease [35,36], and intensive efforts were then made to evaluate the efficacy of these inhibitors in vivo and in clinical trials. Unfortunately, most of these investigations failed to prove the efficacy of those studied drugs in vivo due to problems of hydrolytic instability, toxicity and adverse side effects [37,38]. To date, only one drug, acetohydroxamic acid, has been clinically approved for the treatment of the *H. pylori* infection through urease inhibition. However, some limitations associated with severe side effects, such as teratogenicity, psycho-neurological, and musculo-integumentary symptoms, have resulted in limited use of this therapy [39,40].

On the other hand, positive efficacy with reduced side effects has been observed with the use of acetohydroxamic acid and antibiotics as a combination treatment [41]. This indicates that the use of urease inhibitors, in combination with antibiotics, might be a useful tool to maximize the treatment course of the disease, while minimizing side effects. Phosphoramidates, another group of urease inhibitors, have also shown effective therapeutic efficacy in vivo. However, this group has not been introduced to the market as effective drugs due to its rapid hydrolysis in the gastric juice of the stomach [20,34].

As it can be seen, crucial work is still needed to modify and prepare several useful drug derivatives, by synthesis or semisynthesis, for the future development of more potent urease inhibitors with better stability, no toxicity and less undesirable side effects, which can then be further investigated in clinical trials.

## 4. Concluding Remarks

Although the treatment of *H. pylori* infection in children has greatly advanced through the use of antibiotic therapy, the side effects and persistence of drug-resistant strains have resulted in the significant and rising burden of the disease worldwide, putting strain on health care resources of countries at all economic levels. Therefore, it has become an urgent concern to find alternative strategies to combat this infection. Urease inhibitors are considered to be useful drugs for the treatment of the *H. pylori* infection in children, but testing of some of these drugs on animal models or humans has been prevented because of their toxicity or instability. This hurdle, which complicates the ability to present the effectiveness of urease inhibitors in comparison with standard treatment (antibiotics plus a proton pump inhibitor), must be one of the first targets to overcome for future drug development.

The use of urease inhibitors in combination therapy with antibiotics and proton pump inhibitors is another potential option for the treatment of *H. pylori* infection in the pediatric population; however, these studies are still limited. This lack of research is potentially due to several reasons, such as the fact that the clinical implications of an *H. pylori* infection in children are unclear during childhood, that the prevalence of infection in Western countries is low (limited access to volunteer participants for clinical trials), that the costs of performing such studies on a large scale, and in developing countries where the prevalence of infection is high, are high, and that facilities to carry out clinical trials studies are not widely available. Therefore, all levels of research, including basic, clinical, and population-level, need continued financial support to facilitate development and implementation of effective urease inhibitors with suitable pharmacokinetics, hydrolytic stability, and free toxicological profiles.
